# Efficient Electrochemical Oxidation of Chloramphenicol by Novel Reduced TiO_2_ Nanotube Array Anodes: Kinetics, Reaction Parameters, Degradation Pathway and Biotoxicity Forecast

**DOI:** 10.3390/ma16113971

**Published:** 2023-05-25

**Authors:** Pengqi Wang, Guangyi Chu, Guangfei Gao, Fengchun Li, Yi Ren, Yue Ding, Yawei Gu, Wenqiang Jiang, Xuan Zhang

**Affiliations:** 1 School of Environmental Science and Engineering, Qilu University of Technology (Shandong Academy of Sciences), Jinan 250353, China; 10431201083@stu.qlu.edu.cn (P.W.); 10431201069@stu.qlu.edu.cn (G.G.); 10431211182@stu.qlu.edu.cn (F.L.); 10431201058@stu.qlu.edu.cn (Y.R.); 201993030074@stu.qlu.edu.cn (Y.D.); gyw@qlu.edu.cn (Y.G.); 280530@qlu.edu.cn (W.J.); 2 Jinan Water & Wastewater Monitoring Center, Jinan 250353, China; 13356691767@163.com

**Keywords:** self-supported Ti^3+^-doped TiO_2_ nanotube array, chloramphenicol, electrochemical advanced oxidation, degradation mechanism

## Abstract

The key component of electrochemical advanced oxidation technology are high-efficiency anodes, and highly efficient and simple-to-prepare materials have generated a lot of interest. In this study, novel self-supported Ti^3+^-doped titanium dioxide nanotube arrays (R-TNTs) anodes were successfully prepared by a two-step anodic oxidation and straightforward electrochemical reduction technique. The electrochemical reduction self-doping treatment produced more Ti^3+^ sites with stronger absorption in the UV-vis region, a band gap reduction from 2.86 to 2.48 ev, and a significant increase in electron transport rate. The electrochemical degradation effect of R-TNTs electrode on chloramphenicol (CAP) simulated wastewater was investigated. At pH = 5, current density of 8 mA cm^−2^, electrolyte concentration of 0.1 M sodium sulfate (Na_2_SO_4_), initial CAP concentration of 10 mg L^−1^, CAP degradation efficiency exceeded 95% after 40 min. In addition, molecular probe experiments and electron paramagnetic resonance (EPR) tests revealed that the active species were mainly •OH and SO_4_^−^, among which •OH played a major role. The CAP degradation intermediates were discovered using high-performance liquid chromatography-mass spectrometry (HPLC-MS), and three possible degradation mechanisms were postulated. In cycling experiments, the R-TNTs anode demonstrated good stability. The R-TNTs prepared in this paper were an anode electrocatalytic material with high catalytic activity and stability, which could provide a new approach for the preparation of electrochemical anode materials for difficult-to-degrade organic compounds.

## 1. Introduction

Both in human and animal illness prevention and treatment, antibiotics are frequently employed. However, only a small portion can be absorbed by the organism, and more than 85% of antibiotics are discharged into municipal wastewater in the form of prodrugs or metabolites [1,2]. Traditional wastewater treatment facilities are less effective in removing such substances. The incompletely degraded antibiotics can re-enter the environment through wastewater treatment plant effluent, slowly build up there, and even enrich in the human body through the biological chain, potentially posing a risk to human health [3].

Chloramphenicol (CAP) is a widely used broad-spectrum antibiotic that has great antibacterial activity [4]. However, CAP is possibly carcinogenic and genotoxic to humans, and it could cause aplastic anemia and neurological damage [5]. CAP is currently prohibited in many countries, but due to its low cost and ease of access, it is still widely used in livestock and aquaculture in some low-income nations [6], leading to detectable concentrations of CAP in environmental water bodies [7]. Therefore, it is crucial to find a method that could efficiently remove CAP from water bodies.

Many techniques have been used for CAP removal, Appavu et al. developed BiVO_4_/N-rGo, and under the ideal conditions (CAP concentration of 19.4 mg L^−1^, catalyst concentration of 50 mg L^−1^, and 500 W tungsten filament lamp as the light source) for 300 min, the degradation rate of CAP reached 93%. The reaction rate constant was determined via kinetic fitting to be 0.0091 min^−1^ [8]. Tan et al. successfully prepared nano zero-valent iron-activated PMS for CAP removal, and 95.2% of 10 mg L^−1^ CAP was removed by 0.2 mM peroxymonosulfate and 0.5 g L^−1^ Fe0 at neutral pH, with little Fe^3+^ leaching. The degradation rate of CAP could reach 0.0355 min^−1^ [9]. These approaches, however, had a number of shortcomings. For instance, photocatalysis was limited by its poor efficiency, and nano-zero-valent iron reduction required the addition of supplemental chemical reagents [10].

Compared with the abovementioned methods, electrochemical advanced oxidation processes (EAOPs), which use clean energy, have attracted a lot of attention as an environmentally friendly technology because of their high catalytic efficiency, low cost, and modular application [11,12,13]. EAOPs is divided into two types: Indirect oxidation, which involves the oxidation of water on the anode to produce hydroxyl radicals (•OH), and direct oxidation, which involves the direct transfer of electrons from organics to anode materials [14,15]. Numerous studies have demonstrated that the anode material had a direct impact on the effectiveness of EAOPs [16,17]. The oxidation reaction products, reaction mechanisms, and current efficiencies vary between different anode materials. The development of effective, stable, inexpensive, and simple-to-prepare electrodes has become a bottleneck limiting the further application of EAOPs [18,19].

TiO_2_ has been extensively studied since the discovery of its photocatalytic in 1972 [20,21,22]. Due to TiO_2_’s wide prohibited bandwidth, only 5% of ambient light can be absorbed and used. Fouled water affects the propagation of light in the water body, which, in turn, reduces its light utilization efficiency [19]. TiO_2_ could be employed in electrochemical systems without direct photoactivation to get over these intrinsic obstacles [23]. In comparison to granular and nanorod TiO_2_, nanotubes showed a large specific surface area and might offer more active areas during catalytic processes.

The preparation of TiO_2_ nanotubes using electrochemical anodic oxidation was a self-organizing process that allowed the formation of TiO_2_ nanotubes aligned perpendicular to the Ti surface with a controllable tube length. Without the use of glue or polymers, these nanotubes were attached directly to the Ti surface [22]. The third generation of anodic oxidation technique for making TiO_2_ nanotubes involved applying bias pressure to Ti plates in a solution of ethylene glycol containing fluoride ions (F^−^) to create one-dimensional nanotubes on their surface. Compared to the first generation (electrolyte: Aqueous solution containing HF) and the second generation (electrolyte: Aqueous solution, grown in neutral or buffered electrolytes), the nanotubes obtained using the third generation preparation methods are more ordered and have smoother walls, which implies a larger specific surface area [24]. In previous studies, Li et al. successfully prepared B-doped TiO_2_ nanotubes and demonstrated that the doping of B could further accelerate the degradation rate of phenol [25]. Co-doped TiO_2_ nanotubes were successfully prepared by impregnation, and their service life was significantly increased from 2.3 h to 100 h [19]. Sub-chemometric titanium oxides (Ti_n_O_2n−1_, *n* ≥ 3), i.e., Ti_4_O_7_, Ti_5_O_9_ and Ti_6_O_11_, could be obtained by H_2_ reduction [26]. According to Zaky and Chaplin [27], the Ti_4_O_7_ electrode performed better than the traditional Pt anode and dimensionally stable anodes [28]. However, the harsh conditions for preparation required high temperatures of 1000 °C. In contrast, the electrochemically induced Ti^3+^ self-doped TiO_2_ nanotube preparation process required mild conditions by inducing inherent vacancies and defects in TiO_2_ crystals without introducing any additional elements. Due to these inherent defects, TiO_2_′s lattice parameters changed, changing the energy band structure, and resulting in a narrower forbidden bandwidth. At present, there are few studies on the use of such electrodes to degrade antibiotic wastewater [29,30].

In this paper, the following task are completed: (1) To prepare R-TNTs using a two-step anodic oxidation combined with a simple electrochemical reduction process and analyze the effects of the reduction treatment on R-TNTs, (2) to examine the electrochemical degradation performance of CAP on R-TNTs electrodes, (3) to clarify the active species present in the system using EPR and molecular probe experiments, (4) to reveal CAP degradation pathways and assess the corresponding detoxification in this system.

## 2. Materials and Methods

### 2.1. Experimental Materials

Methanol and triethylamine were chromatographically pure, and all other chemical reagents were analytically pure. All the used chemicals were purchased from Shanghai Maclin Biochemical Technology Co., LTD, Shanghai, China. The experiment’s solutions were made with deionized water. Ti plates with dimensions of 50 × 30 × 1 mm, were purchased from Anhui Xinfeng Technology Co., LTD (Hefei, China).

### 2.2. Experimental Methods

#### 2.2.1. Preparation of TNTs and R-TNTs

The Ti plate was first sanded with 240#, 400#, 600#, 800# sandpaper in sequence to remove any TiO_2_ that might be present on the surface. After that, the grease was removed using 15 min of ultrasonic cleaning with acetone, anhydrous ethanol, and deionized water, respectively. The plates were chemically polished in HF/HNO_3_/H_2_O (1:4:5, *v*/*v*/*v*) mixed solution until their surface took on a metallic shine and were thoroughly rinsed three times with 500 mL deionized water.

Titanium dioxide nanotube arrays (TNTs) were prepared using a two-step anodization process. For the first anodizing, pretreatment Ti plate was used as anode, stainless steel plate of the same size was used as cathode, plate spacing was 1 cm, and the electrolyte was glycol containing 3 wt% NH_4_F and 5 wt% H_2_O at 42 V constant pressure reaction for 4 h. The second anodizing was performed in ethylene glycol solution containing 1 wt% NH_4_F and 1 wt% H_2_O at 42 V constant pressure for 1 h to obtain a better morphology of the nanotubes [31]. Amorphous TNTs were obtained by a two-step anodic oxidation process. After cleaning with deionized water and drying in the air for 15 min, the amorphous TNTs were annealed in a muffle furnace at 450 °C for 2 h, with the heating rate set at 4 °C min^−1^. After that, the TNTs were allowed to cool naturally to room temperature. The resulting electrodes were labeled as TNTs.

R-TNTs were obtained by electrochemical reducing TNTs. It was obtained by electrochemical reduction in a solution of 1 M ammonium sulfate solution for 11 min, using the prepared TNTs as the cathode and a Pt sheet of the same size as the anode at a current density of 2 mA cm^−2^. Finally, deionized water was used to clean, and air dry the R-TNTs.

#### 2.2.2. Electrocatalytic Degradation of CAP

**Figure 1:**
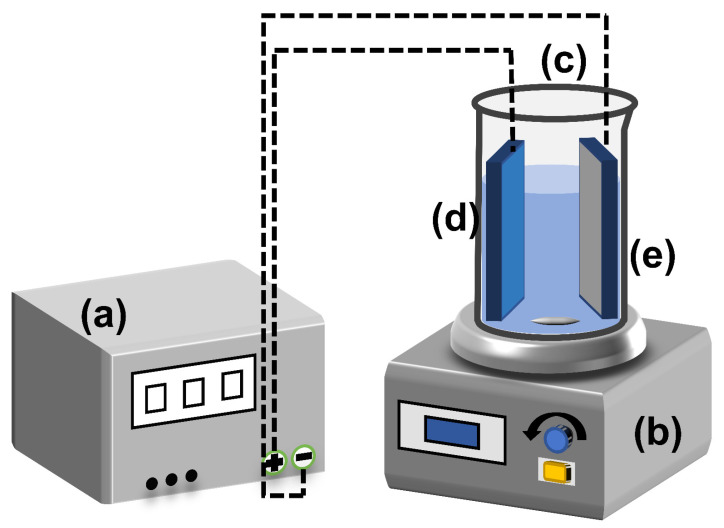
Diagram of experimental apparatus for electrochemical catalysis. (**a**) DC Power, (**b**) thermostatic magnetic stirrer, (**c**) beaker, (**d**) R-TNTs, and (**e**) Ti plate.

The electrolytic cell used in the CAP electrocatalytic tests was a 200 mL beaker with a bottom-mounted magnetic stirrer rotating at a predetermined speed of 500 rpm (Figure 1). During the electrolysis experiment, the temperature was held at room temperature. The prepared electrodes of R-TNTs were used as anodes, and the cathodes were Ti plates of the same size. DC power (PS-303D, Zhao Xin, China) supply supplied a continuous current while the electrode spacing was fixed to 1 cm. At an electrolyte concentration of 0.1 M sodium sulfate, the current density (2, 4, 6, 8, 10 mA cm^−2^), pH (3, 5, 7, 9, 11), and initial CAP concentration (5, 10, 15, 20, 30 mg L^−1^) were all controlled in batches. Each CAP degradation experiment lasted one hour, during which time 0.9 mL of solution was drawn out and placed into a 1.5 mL sample tube that had already been pre-filled with 0.2 mL of methanol. The samples were filtered before each measurement of the CAP concentration using a 0.45 um filter membrane. The initial pH was not adjusted for all experiments except for the experiments examining the impact of pH. The details of the HPLC detection and HPLC-MS analysis of the byproducts of CAP degradation were presented in Texts S1 and S2, respectively. Text S3 contained information about the anode’s electrochemical characterization in more detail.

### 2.3. Physical and Chemical Property Analysis

To characterize the morphology and microstructure of the samples, scanning electron microscopy (SEM) and energy dispersive spectroscopy (EDS) were utilized. X-ray diffraction (XRD) was applied to examine the crystal structure of the samples. A tiny confocal Raman spectrometer was used to take Raman measurements. The valence bands of the TNTs produced and the surface composition of the materials were analyzed using X-ray photoelectron spectroscopy (XPS). Furthermore, a spectrophotometer was used to measure the diffuse reflectance UV-vis spectra. More specific characterization parameters are in the Supplementary Materials (Text S4).

## 3. Results

### 3.1. Electrode Surface Morphology and Structure

**Figure 2:**
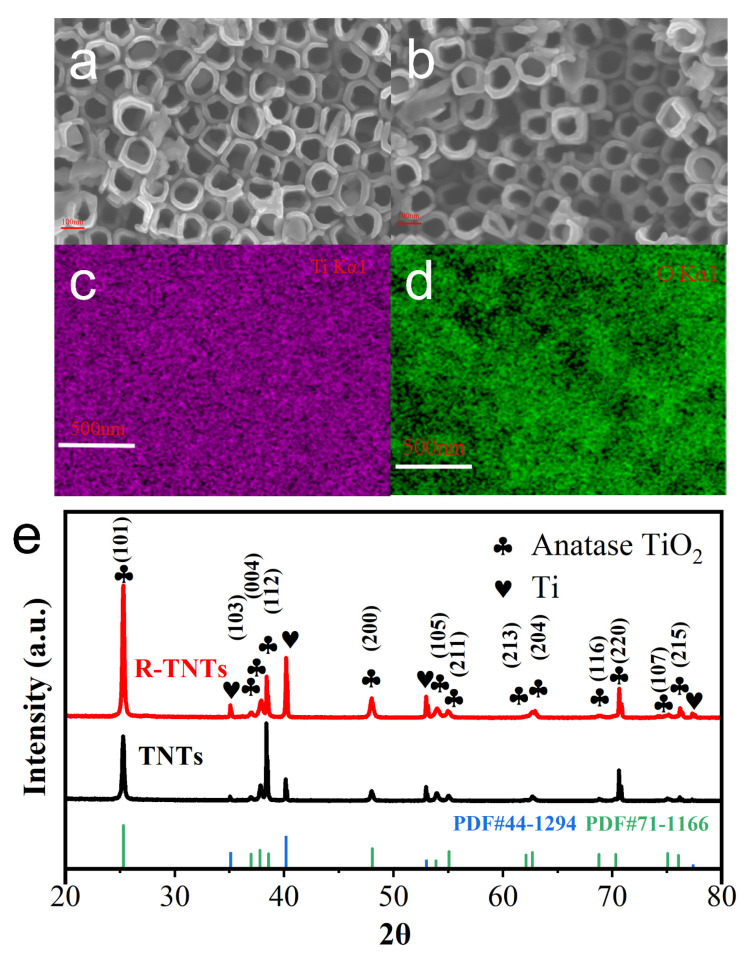
SEM images of (**a**) intact TNTs and (**b**) R-TNTs. Elemental mapping of (**c**) Ti and (**d**) O for R-TNTs and (**e**) XRD patterns.

TNTs prepared by anodic oxidation had a uniform array of nanotubes on their surface (Figure 2a). The surface morphology of TNTs after electrochemical reduction is shown in Figure 2b. The nanotube structure was still present, indicating that the porous structure was not destroyed by electrochemical reduction. However, some of the nanotube walls were corroded, and the gaps between intact nanotubes disappeared. This phenomenon was also seen by Xu et al. [32]. The appearance of some lumps on the top of the nanotubes might be caused by the use of fresh electrolytes [22]. In addition, as shown in Figure 2c,d, Ti and O elements were evenly distributed on the surface of R-TNTs, according to an EDS analysis. R-TNTs’ dominant diffraction peaks matched those of metallic Ti (JCPDS file NO. 44-1294) and anatase TiO_2_ (JCPDS file NO. 71-1166), respectively (Figure 1e), indicating the successful preparation of TNTs on Ti substrates using a two-step anodic oxidation method. The sharp diffraction peaks indicate high crystallization of R-TNTs.

**Figure 3:**
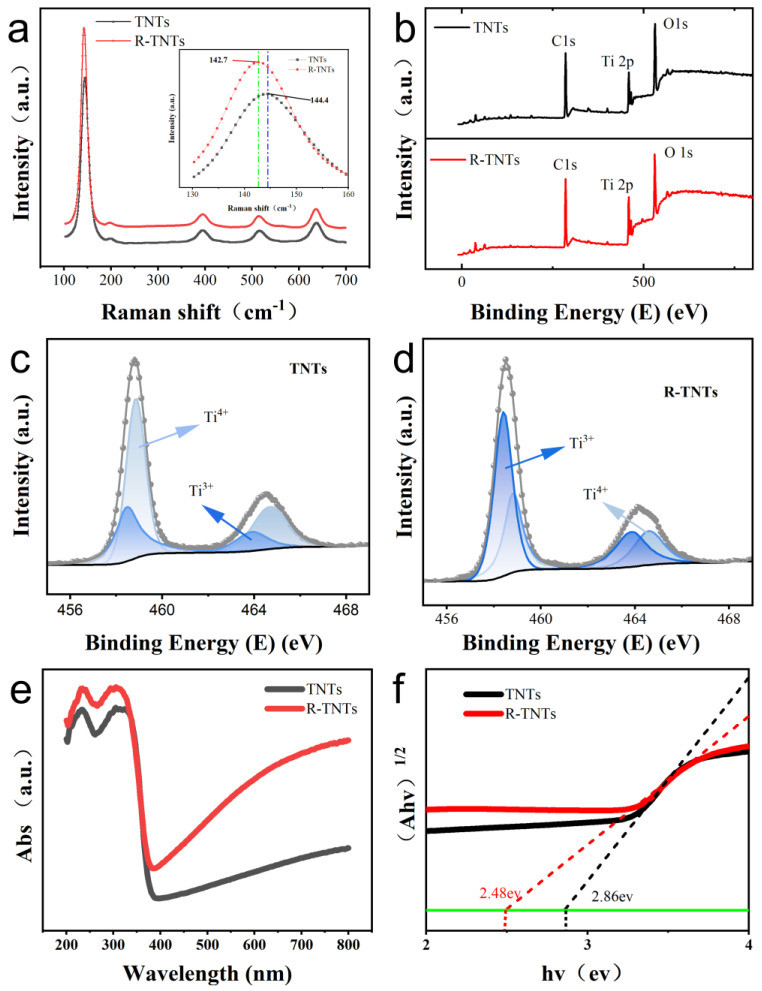
(**a**) Raman spectra of TNTs and R−TNTs. The inset shows the corresponding main Eg Raman mode of samples. (**b**) XPS spectra of TNTs and R−TNTs. Ti 2p XPS spectra of (**c**) TNTs and (**d**) R−TNTs. (**e**) UV−vis absorption spectra of TNTs and R−TNTs (**f**) Tauc plot as a function of the photon ene.

The diffraction peak patterns of R-TNTs showed little difference from those of TNTs (Figure 3a), indicating that the crystal structure remained constant both before and after reduction. Each sample contains five well defined peaks at 144 cm^−1^ (Eg), 198 cm^−1^ (Eg), 395 cm^−1^ (B1g), 517 cm^−1^ (B1g), and 637.7 cm^−1^ (Eg) indicating that TNTs and R-TNTs were both typical anatase phases [33]. With respect to the two Raman spectra, however, they exhibit some differences. In Figure 3a, the primary Eg mode, which was centered at 144 cm^−1^, appeared red-shifted following reduction. The dominant Eg mode was red-shifted as a result of Ti^3+^ self-doping, which caused nonstoichiometry or grains of finite size and enabled phonon confinement [34]. This has been confirmed [32,35].

In order to formally validate the existence of Ti^3+^ in R-TNTs, XPS spectra were analyzed. The binding energy of the sample was corrected to obtain a binding energy of 284.5 eV for C1s. The peaks of Ti, O, and C can be observed in the XPS spectra of both TNTs and R-TNTs, and no peaks of other elements were present (Figure 3b), indicating that no impurities were introduced during the preparation process. The XPS spectra of the corresponding normalized Ti 2p energy levels are shown in Figure 3c. The presence of two broad peaks in the TNTs and R-TNTs that corresponded to the different peaks of Ti 2p_1/2_ and Ti 2p_3/2_ was clearly seen. The peaks of R-TNTs in Figure 3d appeared red-shifted in comparison to TNTs, indicating that their bonding environments were different. The peaks with low binding energies at 458.5 and 463.9 eV could be attributed to Ti^3+^, and the peaks with centers at 458.8 and 464.7 eV were confirmed to belong to Ti^4+^ when the XPS spectra were fitted. The Ti^3+^ content in R-TNTs was elevated by two times compared to TNTs (from 6.9% to 14.2%), indicating that more Ti^3+^ sites were produced by R-TNTs during the process of electrochemical reduction.

According to diffuse reflectance spectra for the UV-vis (Figure 3e), R-TNTs had stronger adsorption than TNTs, which was consistent with earlier research [27]. The surface disorder and the presence of Ti^3+^ defects in R-TNTs were due to their stronger visible and infrared adsorption, which also leads to a continuous vacancy band in the electronic state [32]. The introduction of Ti^3+^ by oxygen vacancies and self-doping reduced the band gap of R-TNTs by 0.38 eV (Figure 3f).

**Figure 4:**
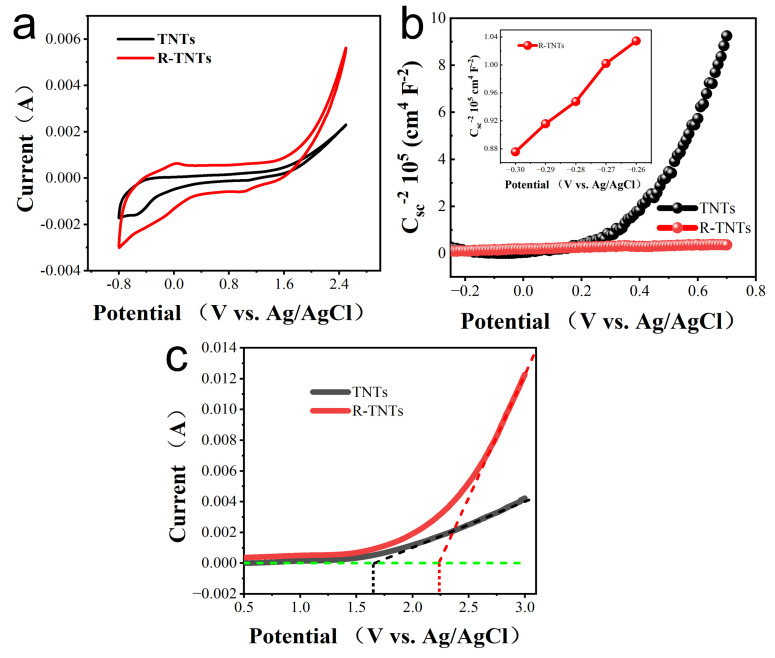
(**a**) CV, (**b**) Mott–Schottky and (**c**) LSV of TNTs and R−TNTs.

The R-TNTs exhibited a better current response, as shown in Figure 4a, and their reduction peak, which formed around −0.45 V, was caused by the reduction of Ti^4+^ to Ti^3+^, demonstrating that some Ti^4+^ was converted to Ti^3+^ during the electrochemical reduction process. The insertion of H^+^ led to a loss of charge [36]. In Figure 4b, the Mott–Schottky plots of TNT and R-TNT are shown. R-TNTs were still n-type semiconductors, as demonstrated by the positive slope of the linear. According to estimates, the E*_fb_* values of TNTs and R-TNTs in comparison to SCE were −0.27 and −0.39 V, respectively (Text S4) [37]. These findings support the hypothesis that electrochemical reduction considerably improves electron transport, increases the conductivity of R-TNTs, and prevents the complexation of valence band holes and conduction band electrons [38].

The side reaction of oxygen evolution frequently happened during the electrocatalytic oxidation of contaminants. The oxygen evolution potential (OEP) of several materials was investigated by LSV. The current efficiency could rise because with a higher OEP value [39]. As shown in Figure 4c, the OEP of R-TNTs was 2.25 V (vs. SCE), which was much higher than that of TNTs at 1.45 V (vs. SCE). The main oxygen precipitation side reactions were successfully reduced by the higher OEP of R-TNTs, increasing the anodic oxidation efficiency.

### 3.2. Electrochemical Degradation of CAP

#### 3.2.1. Influence of Anode Materials

**Figure 5:**
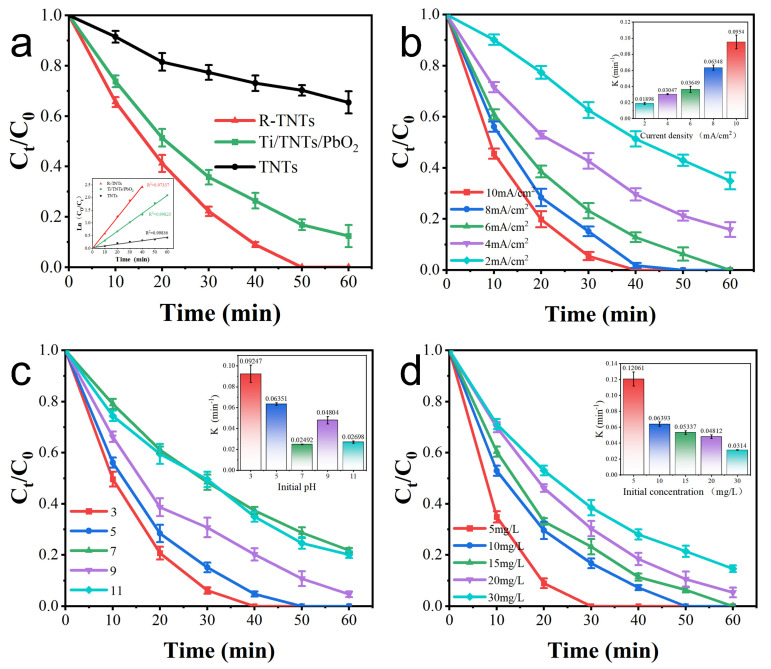
Effects of the (**a**) different anode, (**b**) current density, (**c**) initial pH, and (**d**) initial concentration on degradation of CAP.

Degradation experiments with CAP (10 mg L^−1^) were used to evaluate the electrocatalytic efficiency of different anodes. According to Figure 5a, after 40 min of electrolysis, CAP degraded 90.9% as opposed to TNTs and Ti/TNTs/PbO_2_ anodes, which degraded 26.9% and 73.5%, respectively. According to quasi-level kinetics, CAP degraded, and the degradation rate constant (k*_CAP_*) of the R-TNTs electrode (5.9 × 10^−2^ min^−1^) was 1.7 and 8.7 times higher than that of the Ti/TNTs/PbO_2_ (3.5 × 10^−2^ min^−1^) and TNTs (6.8 × 10^−3^ min^−1^) electrodes, indicating that the electrocatalytic performance of R-TNTs could be significantly enhanced by a straightforward electroreduction.

#### 3.2.2. Effect of Current Density

It was believed that the electrochemical oxidation reaction was greatly influenced by current density [40]. R-TNTs electrode was used to test the impact on the CAP removal ratio at various current densities, pH = 5, and initial concentrations of 10 mg L^−1^. The CAP degradation ratios increased with higher current density, as seen in Figure 5b and inset. At 2, 4, 6, 8 mA, and 10 mA cm^−2^, the CAP removal ratios were 37.4%, 57.3%, 76.8%, 84.9%, and 94.5%, with rate constants of 0.01898, 0.03047, 0.03649, 0.06348, and 0.0954 min^−1^.

The result proves that the current density increase promotes the formation of conduction band electrons and holes, increasing the production of •OH and resulting in higher CAP degradation ratios [41]. According to the research authored by Palma-Goyes [42], excessive current density somewhat increases the oxygen precipitation side reaction while reducing current efficiency. Therefore, we selected a reaction current density of 8 mA cm^−2^.

#### 3.2.3. Effect of Initial pH

Since protons can be involved in many free radical chain reactions, solution pH emerged as one of the key variables influencing the characteristics of electrochemical processes [43]. The effects of pH (3, 5, 7, 9, 11) on CAP degradation was investigated using an initial CAP concentration of 10 mg L^−1^ and a current density of 8 mA cm^−2^. pH was changed in the solution using 0.1 M H_2_SO_4_ and NaOH. As can be seen in Figure 5c, the degradation ratio of CAP rose from 51.5% to 84.9% at 30 min, when the pH was decreased from 7 to 5, and further increased when the pH was decreased to 3. The degradation efficiency at pH 11 was not significantly different from that under neutral conditions (pH = 7) with k values of 0.02698 and 0.02492 min^−1^, respectively. When the pH was alkaline, such as pH = 9, the degradation ratio was 69.24% at 30 min.

Overall, CAP degraded more quickly in acidic conditions than it did under basic conditions. Later explanations for this occurrence included the following: (1) This was due to that an acidic pH promoted CAP to adhere to the surface of R-TNTs, which inhibited the formation of the electron-hole complex and encouraged the enhanced breakdown of CAP. (2) Since •OH had a larger oxidation potential under acidic circumstances (+2.85 V) than under alkaline ones (+2.02 V), low pH prevented the oxygen precipitation process and increased the efficiency of CAP degradation [44]. According to earlier research, CAP has a pKa value of 9.4, which means that at a pH of 9, 50% of CAP is in the form of anions, which are more vulnerable to attack and mineralization by electrophilic free radicals like hydroxyl radicals in solutions [45]. The opposite is accurate with a pH of 11. This explains the variance in CAP degradation rate under different alkaline conditions. R-TNTs are highly applicable as electrodes and can achieve good CAP degradation in a wide pH range.

#### 3.2.4. Effect of Initial CAP Concentration

At pH 5 and 8 mA cm^−2^ of current density, the impact of initial CAP concentration on its degradation was examined. As the initial CAP concentration increased in Figure 5d, the degradation ratio gradually reduced. At the initial concentrations of 5, 10, 15, 20, 30 mg L^−1^, the rate constants of CAP were 0.12061, 0.06393, 0.05337, 0.04862, and 0.0314 min^−1^, respectively. When the initial concentration of CAP was less than 15 mg L^−1^, it could be totally broken down in 60 min, but when it was greater than 20 mg L^−1^, it could not be entirely broken down. This might be due to the fact that at higher initial CAP concentrations, the intermediates generated increased, and they would compete with CAP for active sites, thus affecting the degradation efficiency.

The increase in initial CAP concentration was followed by an increase in CAP degradation amount, which was due to the increase in CAP mass transfer rate as the initial concentration increased (Figure S3). At higher initial concentrations, more CAP reached the electrode surface at the same time to undergo degradation reactions [46]. Under this electrolysis condition, when the initial concentration did not exceed 30 mg L^−1^, enough •OH was generated to react with the CAP reaching the electrode surface for degradation, indicating a diffusion-controlled reaction stage at this stage. If we continued to increase the initial reactant concentration, there were enough CAP reaching the electrode surface per unit time, and the amount of •OH generated at this time was insufficient. In such a case, the reaction rate was determined by the amount of •OH, which was controlled by the current density. The initial concentration of CAP did not reach this state during the course of our experiments.

### 3.3. Electrode Repeatability

**Figure 6:**
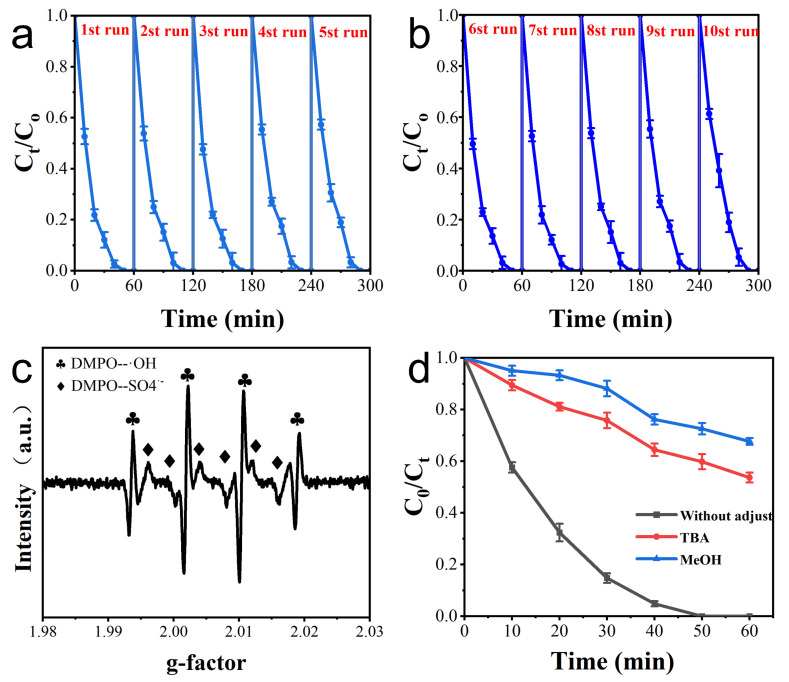
(**a**) CAP degradation and for 5 consecutive experiments using R-TNTs. (**b**) CAP degradation on R−TNTs after regeneration. (**c**) EPR test. (**d**) Queching study on CAP degradation. Conditions: CAP (10 mg L^−1^), current density 8 mA cm^−2^, pH 5, Na_2_SO_4_ 0.1 M. MeOH 5 M, TBA 2.2 M.

The electrode experiment was carried out five times, each time, in a beaker with 200 mL of the solution. The solution was electrochemically degraded at 10 mg L^−1^ CAP, 0.1 M Na_2_SO_4_, pH = 5, and 8 mA cm^−2^ for 1 h. The CAP degradation during the five cycles is shown in Figure 6a. The CAP was completely degraded by electrochemical treatment over 50 min for each of the five cycles. The CAP degradation rate was 87.9% in the first cycle and fell to 81.0% in the fifth cycle if the electrochemical treatment lasted 30 min. The degradation rate of CAP remained stable during five cycles, indicating that R-TNTs had stable electrochemical catalytic degradation performance. The R-TNTs used five times were subjected to reduction treatment before being put to use in more CAP degradation tests. The degradation rates of CAP were 86.4% and 81.0% for the sixth and tenth electrochemical degradation for 30 min, respectively, showing that the reduction treatment restored the electrochemical catalytic activity of R-TNTs (Figure 6b).

### 3.4. Assessment of Main Active Species

Whenever CAP was electrochemically degraded with R-TNTs as the anode and Na_2_SO_4_ as the electrolyte, EPR measurements show that both •OH and SO_4_^•−^ radicals were present (Figure 6c). Free radical scavenging studies were used to evaluate their impact on the electrochemical breakdown of CAP. Methanol (MeOH) reacts with •OH and SO_4_^•−^ at rates of 9.7 × 108 M^−1^ s^−1^ and 1.0 × 107 M^−1^ s^−1^, respectively, which are not significantly different from one another. Tert-butyl alcohol’s (TBA) reaction rates with •OH were (3.8–7.6) × 10^8^ M^−1^ s^−1^, compared to only (4.0–9.1) × 10^5^ M^−1^ s^−1^ with SO_4_^•−^ [47]. Thus, MeOH could completely capture both •OH and SO_4_^•−^ radicals, while TBA would preferentially capture •OH, and SO_4_^•−^ retained in solution could continue to react with organic matter.

The removal ratio of CAP at 60 min decreased to 32.33% and 46.31%, respectively, when MeOH and TBA were added to the reaction system, as shown in Figure 6d, demonstrating that •OH was a significant contributor. After MeOH was used to capture both •OH and SO_4_^•−^, the removal ratio of CAP could still reach 32.33%, and this portion of CAP degradation was due to direct oxidation occurring at the electrode surface.

### 3.5. Electrochemical Degradation Mechanism of CAP and Toxicity Analysis

**Figure 7:**
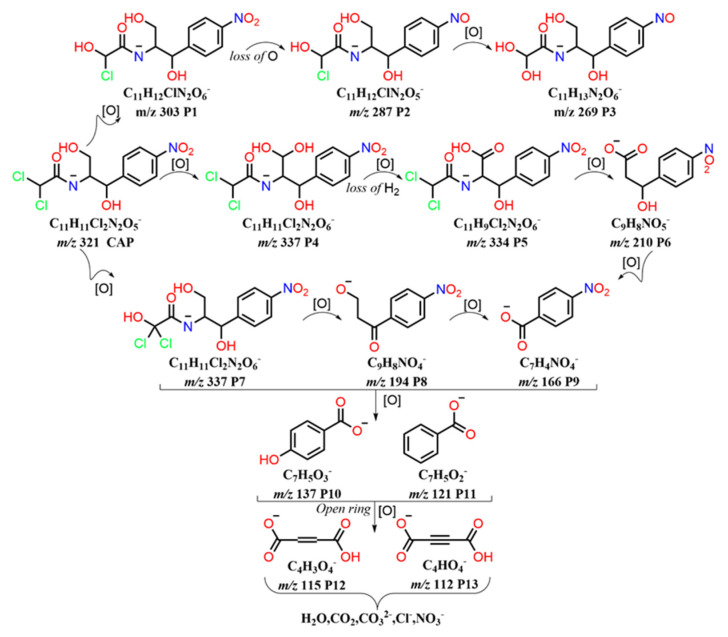
CAP degradation products and pathways inferred from HPLC−MS.

Propylene glycol, dichloroacetamide, and the nitrobenzene ring compensate for the chemical structure of CAP. Figure S4 displays the intermediate products that were detected by HPLC-MS. The Bond Dissociation Energies (BDEs) theory claims that the chemical bond becomes more active as BDE decreases, making it easier for electrons to be lost and for reacting substances to attack it [48]. Phenyl-nitro, O-H, and C-Cl bonds in CAP molecules have low dissociation energies, making them easily cleavable [49]. According to the results of the experiments and the theory of BDEs, mineralization, ring opening, and free radical processes may be potential electrochemical breakdown mechanisms for CAP. Figure 7 depicts the three potential paths for CAP degradation.

The dechlorination and subsequent hydroxylation of CAP were implicated in the first degrading reaction pathway, which gave rise to the product P1 (*m*/*z* 303), whose best-fitting formula was C_11_H_12_ClN_2_O_6_^−^ (deprotonated molecule). The oxygen atom on the nitro group was deoxygenated to form the nitroso product P2 (*m*/*z* 287), then a second chlorine atom was dechlorinated and hydroxylated to produce the product P3 (*m*/*z* 269).

The second degradation reaction chain mainly includes hydroxylation and oxidation reactions. The P4 yielded an *m*/*z* ratio of 337, for which the best-fit formula was C_11_H_11_Cl_2_N_2_O_6_^−^ (deprotonated molecule). This species corresponds to the addition of a hydroxyl radical to CAP, consistent with the formation of a monohydroxylated derivative, as observed in the literature [50]. The additional product P5 (*m*/*z* 334) is produced in the hydroxyl radical-dominated process of oxidative dehydrogenation. Following further oxidation, dichloroacetamide is removed, leaving the product P6 (*m*/*z* 210). This product was vulnerable to •OH attacks on carbon atoms near the phenyl group, resulting in unstable germinal diol, which was ultimately transformed into nitrobenzoic acid P9 (*m*/*z* 166).

The hydroxylation of the terminal carbon that contains two chlorine atoms is the third possible reaction pathway. This process produces the product P7, with an *m*/*z* ratio of 337, in which the most suitable molecular formula is C_11_H_11_Cl_2_N_2_O_6_^−^ (deprotonated molecule), which leads in a 16-unit increase in the mass of the CAP. Further oxidation leads to C-N bond fracture and removal of dichloro-hydroxy-acetamide to produce P8 (*m*/*z* 194). This product has also been reported in the past, and P8 was explained by the synthesis of benzene carbon groups and the loss of acyl amino groups in CAP [51]. P9 (*m*/*z* 166) was created through further oxidation and could alternatively be created from P6 (*m*/*z* 210).

The breakdown of the benzene ring could create tiny acid molecules, such as butynedioic acid P12 (*m*/*z* 115) and butenedioic acid P13 (*m*/*z* 112), which eventually mineralized into H_2_O, CO_2_, NO_3_^−^, Cl^−^, NH_4_^+^, etc., realizing the complete degradation of CAP, was the oxidative degradation of the aforementioned products.

**Figure 8:**
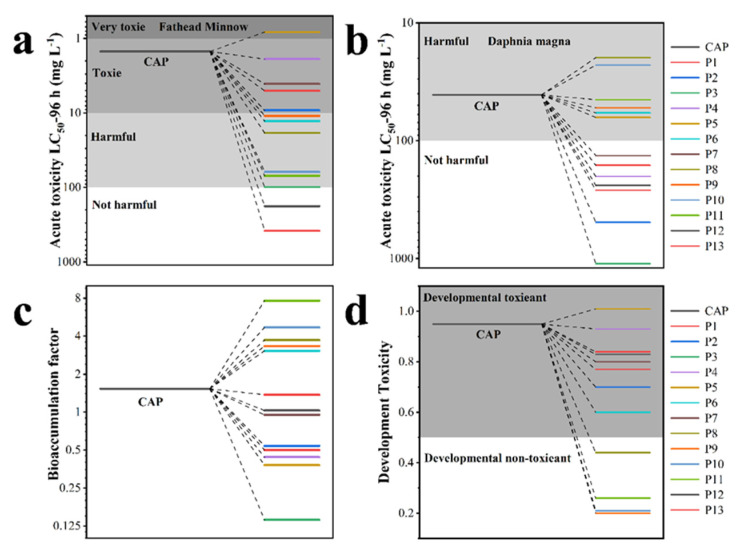
(**a**) Fathead minnow LC50 (96 h), (**b**) daphnia magna LC50 (48 h), (**c**) bioaccumulation factor, and (**d**) developmental toxicity of CAP and its degradation intermediates.

The advantages of the oxidation process can be demonstrated by the analysis of the toxicity of the intermediate products [52]. The acute toxicity of CAP and its breakdown products (Fathead minnow and Daphnia magna), bioaccumulation factor, and developmental toxicity were evaluated using Toxicity Estimation Software (T.E.S.T.), which is based on quantitative structure–activity relationship (QSAR) prediction. For CAP, the fathead minnow’s LD_50_ was 1.49 mg L^−1^, calling it “Toxic” (Figure 8a). In addition, only P5 had LD_50_ values lower than CAP, which was considered “Very Toxic”, while all other intermediates had toxicity lower than CAP. The acute toxicity was greatly decreased with further degradation, and the LD_50_ with the less-toxic compounds was deemed “Not harmful”. Daphnia magna’ s LD_50_ for the acute toxicity of CAP is 40.82 mg L^−1^ (Figure 8b), which is considered to be “Harmful”. Except for P7, almost all of CAP’s degradation intermediates exhibit high LD_50_ values, indicating decreased toxicity of the intermediates, especially for P15. The bioaccumulation factors of various intermediate products were dramatically reduced following treatment using R-TNTs as the anode, with intermediate product P3 having the greatest bioaccumulation factors at 90.8% (Figure 8c). As seen in Figure 8d, after electrocatalytic treatment, all intermediates except P5 had their developmental toxicity decreased, and several of them (P8, P9, P10, and P11) were even discovered in the “Developmental Non-toxic” zone. The outcomes demonstrate that CAP can be fully removed by electrochemical oxidation employing R-TNTs as an anode, as well as effectively reduced toxicity.

## 4. Conclusions

A novel R-TNTs was prepared by a two-step anodic oxidation and electrochemical reduction method. In situ generation of uniform nanotubes on Ti substrates by anodic oxidation treatment and partial conversion of Ti^4+^ ions to Ti^3+^ ions by electrochemical reduction resulted in an increase in UV-visible absorption and a band gap reduction of 0.38 ev. The CAP degradation rate exceeded 95% in 40 min with a reaction rate constant as high as 0.06348 min^−1^, 0.1 M Na_2_SO_4_ as the electrolyte, a current density of 8 mA cm^−2^, pH = 5, and an initial CAP concentration of 10 mg L^−1^. Three potential degradation paths were proposed, and •OH significantly contributed to the CAP degradation procedure. The toxicological simulation revealed that the possible intermediates formed during the CAP degradation exhibited lower toxicity than the pristine. The R-TNTs electrode showed good stability in 5-cycle experiments, and its electrochemical activity could be restored by electrochemical reduction treatment.

## Data Availability

Not applicable.

## Supplementary Materials

The following supporting information can be downloaded at: https://www.mdpi.com/article/10.3390/ma16113971/s1, Text S1: CAP conditions were determined by HPLC; Text S2: Conditions for detection by HPLC-MS; Text S3: Electrochemical test; Text S4: Details of various characterization techniques; Text S5: Calculation formula for electron transfer rate; Figure S1: Formation Mechanism of Anodic TiO_2_ Nanotubes [37,53]; Figure S2: EDS of TNTs and O 1s XPS spectra of (a) TNTs and (b) R-TNTs; Figure S3: The kinetic fit curve of CAP degradation using R-TNTs, (a) current density, (b) initial pH and (c) initial concentration on degradation of CAP; Figure S4: Absolute degradation of different initial pollutant concentrations; Figure S5: The MS of CAP degradation intermediates; Figure S6: The results of liquid mass spectrometry for degradation intermediates of CAP.
