# Supplementary material for: Efficient Electrochemical Oxidation of Chloramphenicol by Novel Reduced TiO2 Nanotube Array Anodes: Kinetics, Reaction Parameters, Degradation Pathway and Biotoxicity Forecast
Source: Materials (Basel). 2023 May 25;16(11):3971. doi: 10.3390/ma16113971 (PMC10254203; doi:10.3390/ma16113971)
Supplement: Supplementary file 1 [file materials-16-03971-s001.zip › materials-2385729-supplementary-new.pdf]

**Supplementary materials**

# Efficient electrochemical oxidation of chloramphenicol by novel reduced $TiO_2$ nanotube array anodes: kinetics, reaction parameters, degradation pathway and biotoxicity forecast

Pengqi Wang [a], Guangyi Chu [b], Guangfei Gao [a], Fengchun Li [a], Yi Ren [a], Yue Ding[a], Yawei Gu [a] , Wenqiang Jiang [a] , Xuan Zhang [a, 1]

[a] School of Environmental Science and Engineering, Qilu University of Technology (Shandong Academy of Sciences), Jinan 250353, China;

[b] Jinan Water & Wastewater Monitoring Center, Jinan 250353, China.

* Corresponding author.

Prof. Xuan Zhang: zx@qlu.edu.cn

[1] *Corresponding author. Tel: +86-152-691--33848. E-mail: zx@qlu.edu.cn (First) Note.

**List of texts**

**Text S1** CAP conditions were determined by HPLC.

**Text S2** Conditions for detection by HPLC-MS.

**Text S3** Electrochemical test.

**Text S4** Details of various characterization techniques.

**Text S5** Calculation formula for electron transfer rate.

**List of Figures**

**Fig. S1.** Formation Mechanism of Anodic $TiO_2$ Nanotubes.

**Fig. S2.** EDS of TNTs.

**Fig. S3.** O 1s XPS spectra of (a) TNTs and (b) R-TNTs.

**Fig. S4.** The kinetic fit curve of CAP degradation using R-TNTs, (a) current density, (b) initial pH and (c) initial concentration on degradation of CAP.

**Fig. S5.** Absolute degradation of different initial pollutant concentrations.

**Fig. S6.** The results of liquid mass spectrometry for degradation intermediates of CAP.

**Supplementary method:**

**Text S1:** CAP conditions were determined by HPLC

The concentration of CAP in the samples was determined using high performance liquid chromatography (HPLC, LC-20ATvp, Shimadzu, Japan), with the wavelength set at 278 nm. The mobile phase was composed of aqueous solution: methanol = 70:30 (v:v), where the aqueous solution was prepared from 0.005 mol $L^{-1}$ sodium heptanesulfonate, 0.05 mol $L^{-1}$ $KH_2PO_4$, and 0.5vt% triethylamine. A pH meter (FE20, Mettler Toledo Instrument Co., Ltd.) was employed to determine pH.

**Text S2:** Conditions for detection by HPLC-MS

HPLC-MS (qtof 6550, Agilent, America) was applied to identify the intermediates resulting from CAP degradation. The injection volume (2 μL), sheath gas flow (12 L $min^{-1}$), sheath gas temperature (350 ℃), column (waters BEH C18) and flow rate (0.3 mL $min^{-1}$) were set, respectively. Formic acid (0.1%) and acetonitrile served as the mobile phases, and the gradient elution settings were 0–2 min, A:B = 5:95, 2–32 min, A:B = 95: 5, and 32–40 min, A:B = 5:95.

**Text S3:** Electrochemical test

A computer-controlled constant potential meter (CHI 760D) was connected to the electrode module. A single-chamber cell with three electrodes was used, consisted of an SCE reference electrode, a working electrode (TNTs or R-TNTs, 1×1 cm) and a Ti plate (1×1 cm) counter electrode. The anode and cathode were separated by a distance of 1 cm and 0.1 M $Na_2SO_4$ was chosen as the supporting electrolyte. With a scan rate of 100 mV $s^{-1}$, cyclic voltammetry (CV) was used over a potential range of -1.0 to +2.7 V (*vs.* SCE). Mott-Schottky experiments were performed at an alternate current (AC) level of 10 mV, a potential range of -0.5 to +0.7 V (*vs.* SCE), and a frequency of 1000 Hz.

**Text S4:** Details of various characterization techniques.

The sample was directly characterized by SEM without gold spraying, and the acceleration voltage was set to 15kv. The X-ray diffraction (XRD) conditions were set as Cu Kα radiation and operated at 40kV/40mA. The scanning interval was 10° ~ 85° and the scanning speed was set as 20° $min^{-1}$.The prepared materials were tested using UV-Visible diffuse reflection spectrometer (UH4100, Hitachi, Japan). Integrating sphere component was used, $BaSO_4$ was used as reference, and the test band was 200-800nm. Raman measurements were performed at room temperature using a Renish modular Raman system with a He-Ne laser 632.8 nm line as the excitation laser, in the range of 100-700 nm.

**Text S5:** Calculation formula for electron transfer rate.

$$\frac{1}{Csc^2} = \left[\frac{2}{e\varepsilon_0\varepsilon N_D}\right]\left[\left(E_S - E_{fb}\right) - \frac{KT}{e}\right] \qquad (1)$$

where Csc is the space charge capacitance ($F/cm^2$); e is elementary charge ($1.602 \times 10^{-19}$ C); ε is the relative dielectric constant of electrode material (48 for anatase TNTs; assumed to be identical for R-TNTs), $\varepsilon_0$ is the permittivity of vacuum ($8.85 \times 10^{-12}\ N^{-1}\ C^2\ /m^2$); $E_S$ is the applied potential (V); $E_{fb}$ is the flat band potential (V); k is the Boltzmann's constant ($1.38 \times 10^{-23}$ J/K), and T is the absolute temperature (K).

**Supplementary Figures**

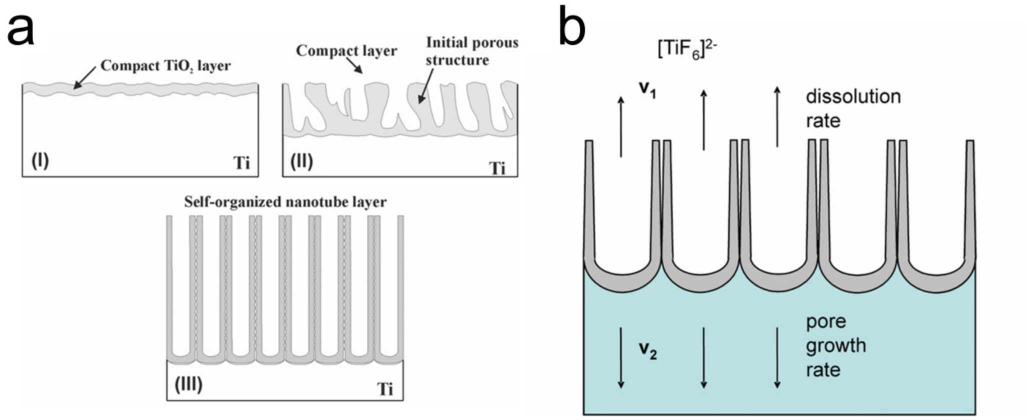


**Figure S1.** (a) The forming process of $TiO_2$ nanotube arrays. The formation of $TiO_2$ nanotube arrays can be divided into three different morphological stages (I–III). (I) A barrier oxide is formed. (II) The surface is locally activated and pores start to grow randomly. (III) Self-organized nanotube layer is formed reproduced. (b) steady state growth situation characterized by equal rates of $TiO_2$ dissolution (v1) and formation (v2)[1].

That is in the process of electrochemical anodization, $TiO_2$ nanotube arrays are formed by self-organization of titania because of three relatively independent procedures: electrochemical oxidation of Ti into $TiO_2$, the electrical field-induced dissolution of $TiO_2$, and the fluorine ion-induced chemical dissolution of $TiO_2$, reaching a delicate balance[1]. Indeed, if the pore initiation phase is followed in a concrete case by SEM images, exactly the sequence described in Fig S1b can be observed[2].

The fact that the layer thickness and the current density reach a limiting value after a certain polarization time can be explained by a steady state situation depicted in Fig S1b[1].

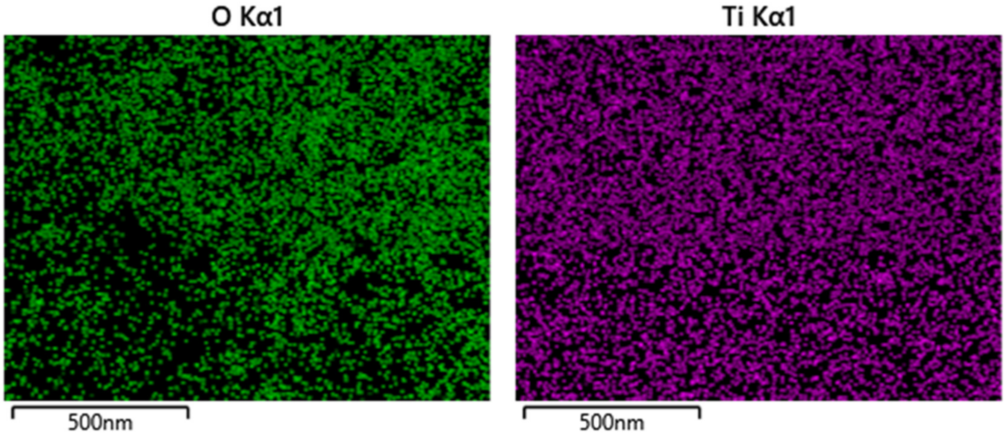


**Figure S2**. EDS of TNTs.

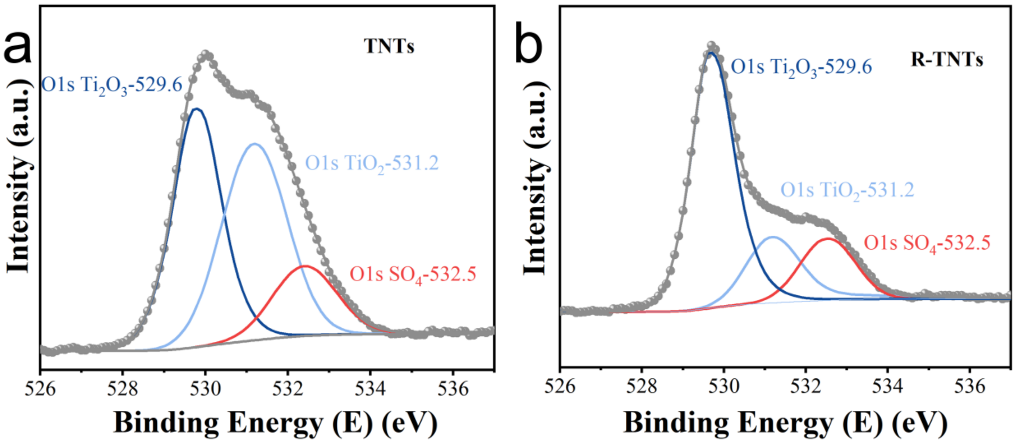


**Figure S3**. O 1s XPS spectra of (a) TNTs and (b) R-TNTs.

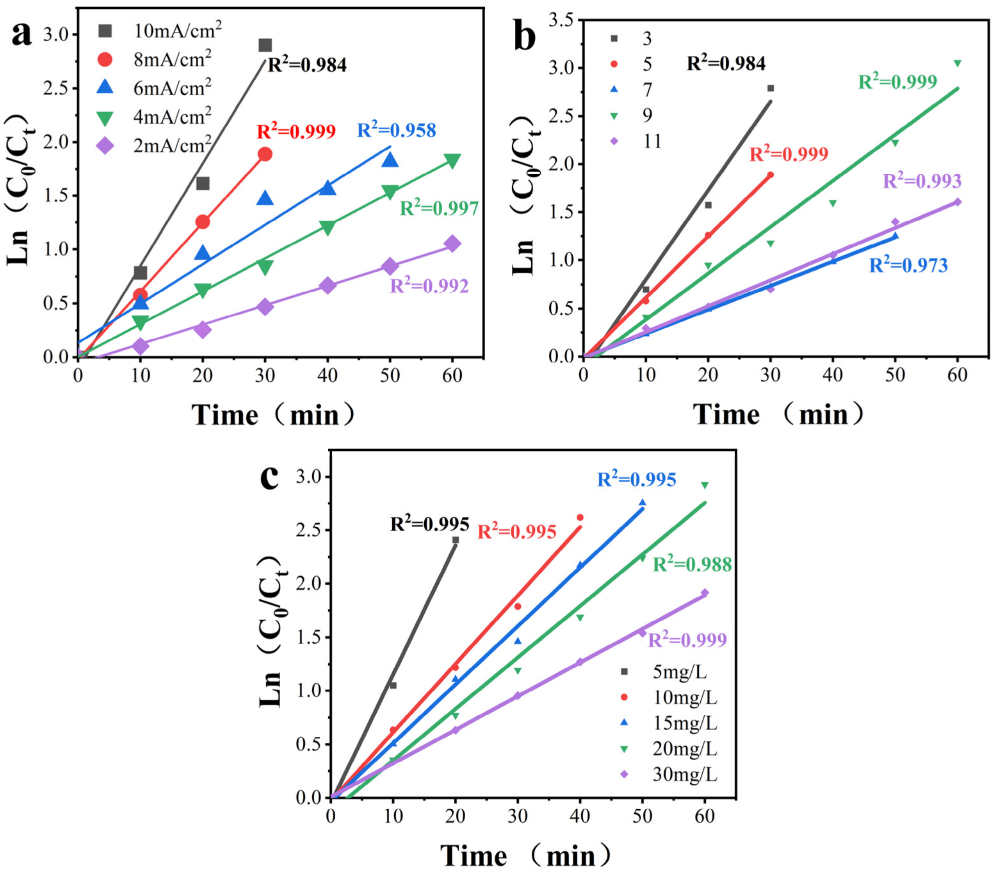


**Figure S4.** The kinetic fit curve of CAP degradation, (a) current density, (b) initial pH and (c) initial concentration on degradation of CAP.

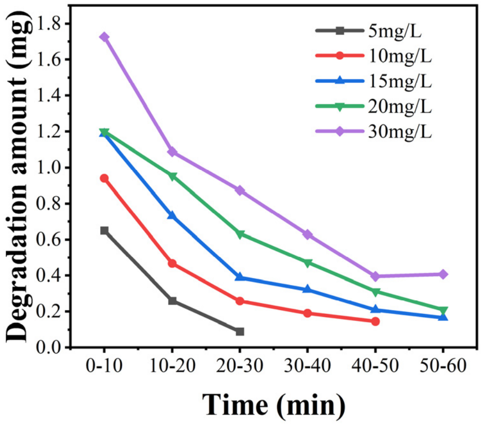


**Figure S5.** Absolute degradation of different initial pollutant concentrations.

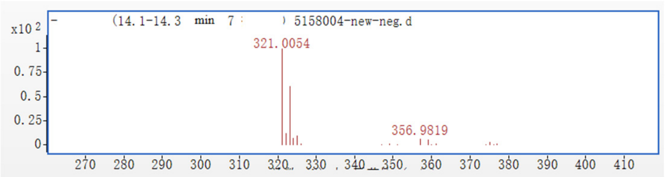
x10 2
(14.1-14.3 min 7
) 5158004-new-neg.d
321.0054
356.9819

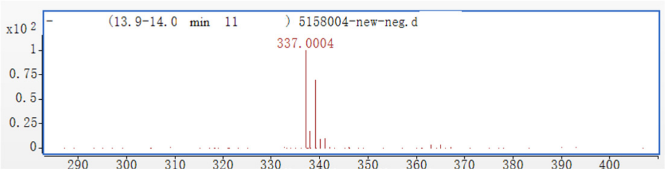
x10 2
(13.9-14.0 min 11
) 5158004-new-neg.d
337.0004

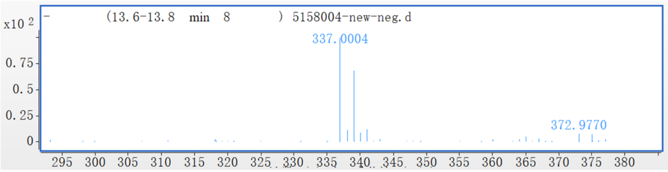
x10 2
(13.6-13.8 min 8
) 5158004-new-neg.d
337.0004
372.9770

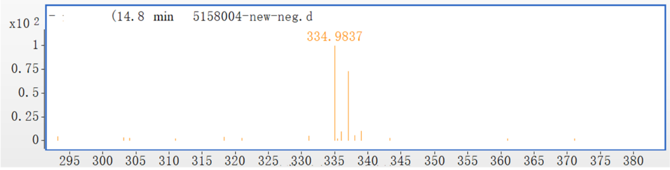
x10 2
(14.8 min 5158004-new-neg.d
334.9837

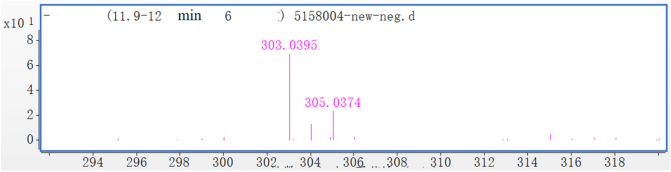
x10 1
(11.9-12 min 6
) 5158004-new-neg.d
303.0395
305.0374

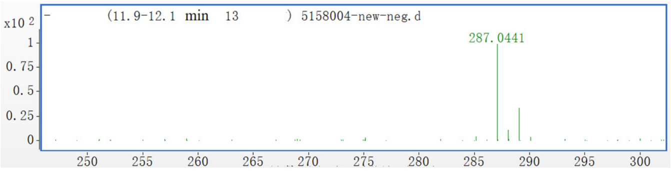
x10 2
(11.9-12.1 min 13
) 5158004-new-neg.d
287.0441

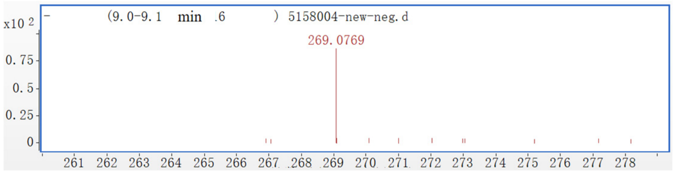
x10 2
(9.0-9.1 min 6
) 5158004-new-neg.d
269.0769

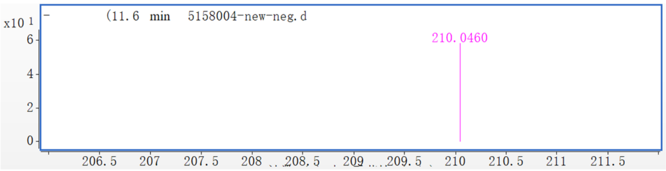
x10 1
(11.6 min 5158004-new-neg.d
210.0460

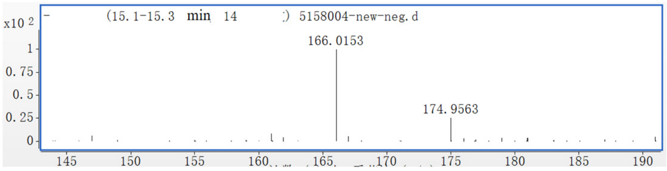
x10 2
(15.1-15.3 min 14
) 5158004-new-neg.d
166.0153
174.9563

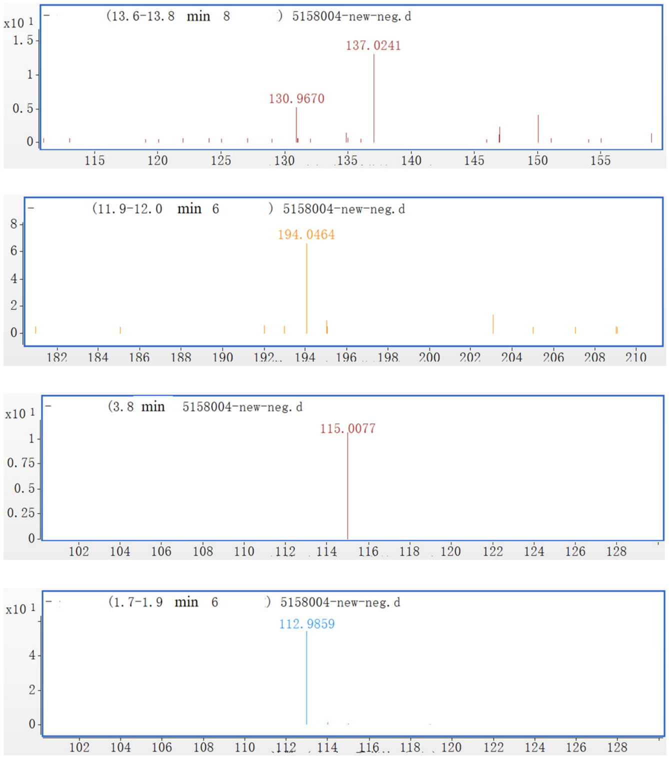


**Figure S6.** The results of liquid mass spectrometry for degradation intermediates of CAP.

1. Macak, J.M.; Tsuchiya, H.; Ghicov, A.; Yasuda, K.; Hahn, R.; Bauer, S.; Schmuki, P. $TiO_2$ Nanotubes: Self-organized Electrochemical Formation, Properties And Applications. *Curr. Opin. Solid State Mater. Sci.* **2007**, *11*, 3-18, doi:10.1016/j.cossms.2007.08.004.
2. Taveira, L.V.; Macák, J.M.; Tsuchiya, H.; Dick, L.F.P.; Schmuki, P. Initiation and Growth of Self-organized $TiO_2$ Nanotubes Anodically Formed in $NH_4F$ / ( NH4 ) $_2SO_4$ Electrolytes. *J. Electrochem. Soc.* **2005**, *152*, B405, doi:10.1149/1.2008980.
